# Pain in Amyotrophic Lateral Sclerosis: A Neglected Aspect of Disease

**DOI:** 10.1155/2011/403808

**Published:** 2011-05-03

**Authors:** Chalonda R. Handy, Christina Krudy, Nicholas Boulis, Thais Federici

**Affiliations:** Department of Neurosurgery, Emory University, 101 Woodruff Circle, Room 6339, Atlanta, GA 30322, USA

## Abstract

Amyotrophic lateral sclerosis (ALS) is a fatal neurodegenerative disorder marked by progressive loss of motor neurons, muscle wasting, and respiratory dysfunction. With disease progression, secondary symptoms arise creating new problematic conditions for ALS patients. Amongst these is pain. Although not a primary consequence of disease, pain occurs in a substantial number of individuals. Yet, studies investigating its pathomechanistic properties in the ALS patient are lacking. Therefore, more exploratory efforts into its scope, severity, impact, and treatment should be initiated. Several studies investigating the use of Clostridial neurotoxins for the reduction of pain in ALS patients suggest the potential for a neural specific approach involving focal drug delivery. Gene therapy represents a way to accomplish this. Therefore, the use of viral vectors to express transgenes that modulate the nociceptive cascade could prove to be an effective way to achieve meaningful benefit in conditions of pain in ALS.

## 1. Introduction

Amyotrophic lateral sclerosis (ALS) is a progressively lethal motor neuron disorder that affects roughly 2 in 100,000 individuals each year [1–3]. Commonly referred to as Lou Gehrig's disease, ALS is characterized by degeneration of primary motor neurons in the cortex, brainstem, and spinal cord. The amyotrophy (atrophy of muscle fibers) leads to muscular paralysis due to loss of innervating motor neurons. The lateral sclerosis typical of the disease refers to the upper motor neuron axonal loss, the hardening of corticospinal tracts, and the resultant gliosis [4, 5]. These changes can lead to a number of debilitating conditions that reflect aberrant functioning in both upper and lower motor neurons. Primary symptoms of ALS include muscle weakness and atrophy, spasticity, speech disturbances, poor management of oral secretions, difficulty in swallowing, and respiratory insufficiencies that usually result in death. These characteristic features of ALS are also accompanied by a number of secondary conditions that can be just as burdensome as those symptoms directly associated with the disorder. Amongst these indirect complications related to the disease is pain. Although not generally associated with ALS, pain has been reported to occur in nearly 70% of ALS patients at some time during the course of the disease [6–8]. Moreover, the frequency of pain seems to be directly proportional to disease progression [7]. Devastatingly, pain is one of the most overlooked, understudied, and poorly managed features of the disorder. No randomized, controlled drug trials have been conducted to investigate pain in ALS, nor have any published observational studies been performed to determine the most effective therapies for ALS pain treatment. Moreover, a recent comprehensive review of ALS literature cited fewer than 10 case series that described drug therapy for ALS pain management [9]. The scarcity of studies directed towards proper pain evaluation and management suggests that in the ALS patient, pain is underrated and could be frequently undertreated, begging the need for more investigations into the prevalence, pharmacological approaches, and pathomechanisms of this important aspect of motor neuron disease.

## 2. Pain in ALS

Pain is described as an unpleasant sensory and emotional experience in response to noxious stimuli, tissue injury, or trauma. Pain can be acute or chronic depending on its duration and the presence of structural and/or functional abnormalities that affect how nerves transmit nociceptive information to the central nervous system [10–13]. 

Although not considered to be a primary consequence of ALS, pain occurs in a substantial number of individuals. Yet, studies investigating the pathomechanistic properties of this condition and the most effective means for achieving nociceptive control in the ALS patient are lacking. Even with the evolution of science regarding potential pain therapeutics, very little effort has been made to understand how these agents are relevant in the context of ALS pain. Pain, therefore, should be recognized as an important aspect of ALS palliative care.

### 2.1. Musculoskeletal Pain

Both transitory acute pain and persistent chronic pain have been reported in ALS. This pain is primarily the result of inactivity and/or the presence of joint inflammation that creates pain at the points of pressure. Pain in ALS most frequently involves musculoskeletal pain that occurs in the back, legs, arms, shoulder, and neck. Although the etiology of this pain is not well understood, it is known that musculoskeletal pain in ALS develops secondary to muscle atrophy and decreased muscle tone. It can be representative of damage to bones, tendons, ligaments, joints, nerves, or the affected muscle itself. An imbalance in this intricate network can greatly affect muscle coordination, strength, and function. It has been documented that muscle denervation, paralysis, and disuse can affect the nerve conduction properties of muscle afferents [14–16]. It also seems likely that chronic muscle wasting in ALS and the resultant pathology could have drastic effects on the nociceptive cascade. 

Muscle denervation is associated with axonal sprouting and an increase in size of surviving motor units [14, 16, 17]. These changes in structural anatomy might produce a pathophysiological condition that results in pain [8]. Thus, one could envision that the series of events that promote the development of musculoskeletal pain in ALS would involve the following steps. Muscle wasting would incite collateral axonal sprouting that enhances the surviving units and creates a larger endplate zone, resulting in less synchronized motor unit action potentials. This would lead to a progressive dissociation of the mechanical and electrical properties of the muscle that worsen over time. This alteration in muscle coordination and force generation properties (onset, amplitude, duration, and polyphasic potentials) causes abnormal stress on the ligaments, tendons, and joints [8, 18–21]. These excessive strains could result in microtrauma to the muscle, resulting in low levels of inflammation that can later have compounding effects due to insufficient healing of the affected tissues. Repetitive bouts of injury due to continual muscle wasting and decreased strength, coordination, and tone can gradually allow for pain development. Moreover, changes in posture, poor body mechanics, and prolonged immobility (all characteristic of ALS) can result in spinal alignment problems and muscle shortening, thereby creating a more painful condition.

### 2.2. Muscle Cramping

Although musculoskeletal pain seems to typically arise during the late stages of ALS, which suggests it is a cumulative event, cramps and fasciculations are more frequent at initial stages. Cramps can be extremely painful and occur in any muscle. Nevertheless, these excruciating conditions are seldom presenting symptoms. In fact, although many patients experience this symptom some months before the onset of muscle weakness, concern regarding these muscle fasciculations is only made after diagnosis [5]. Thus, this is a feature of ALS that is often ignored. Cramping can be exacerbated by cold weather or decreased circulation caused by maintaining the muscle in the same position for an extended period of time. With time, cramps become less severe, however. At later stages of ALS, as the disease progresses to complete paralysis, nerve cells lose the ability to stimulate muscle contractions. 

### 2.3. Spasticity

Spasticity is another common feature of ALS. By definition, spasticity is a velocity-dependent form of hypertonia marked by an increase in tonic stretch reflexes [22, 23]. The hyperactive stretch reflexes associated with spasticity are due to abnormal proprioceptive input in the spinal cord. However, this imbalance in supraspinal inhibitory and excitatory inputs can also perturb the nociceptive reflexes resulting in flexor and extensor spasms [23]. Muscle spasms in ALS are usually due to changes in upper motor neurons of the motor cortex. This distortion in upper motor neuron processing can produce the primitive reflex, Babinski sign, which is one of the most important features of clinical neuropathy [24]. Spasticity itself is not always painful, but it can induce painful cramps, cause muscle fatigue, or alter manual dexterity. Furthermore, spasticity can have musculoskeletal consequences due to involuntary mobilization of stiff joints, muscle contractures, pressure pain, such as shoe agitation due to striatal toe of Babinski sign or even decubitus ulcers due to immobility and skin breakdown in flexor creases [25–28]. All of these muscle hyperactivity-induced changes can distort muscle mechanics to a degree that substantially alter posture, range of motion, ambulation, and gait, thus creating new sources of pain [29].

Although the literature concerning the relationship between stride parameters and nociception is lacking, it has been shown that gait analysis is a useful assessment of function in chronic pain sufferers [30–32]. Changes in gait have been observed in ALS, and alterations of gait dynamics would result from muscle weakness, decreased tone, and endurance as well as alterations in motor cortex excitability, muscle fiber conduction, velocity, and mechanical efficiency [33, 34]. ALS characteristic upper motor neuron pathology can affect all of these factors in addition to promoting spasticity by limiting brainstem control of the vestibulospinal and reticulospinal tracts. 

## 3. Current ALS Pain Therapies

There is no cure for ALS. Likewise, there is no single most effective therapy for ALS-associated pain. Palliative care for ALS patients involves a combinational treatment approach that addresses not only the oropharyngeal, respiratory, nutritional, psychological, and motor functional concerns of the patient, but also the disabling nociceptive features of the disorder as well. 

Again, most of the pain associated with ALS is believed to be due in large part to immobility. Physiotherapy, stretching, and range of motion exercises are used in combination with pharmacotherapies to prevent contractures and reduce cramping, spasticity, and pain. In ALS, routine moderate resistance exercise has been shown to improve static force in muscle groups and slow functional decline. Joint mobilization techniques as well as frequent sustained lengthening of affected muscle groups are also effective in reducing some of the musculoskeletal pain, spasticity, and cramping experienced by ALS patients [35, 36]. 

Drug therapies administered to ALS sufferers early on in the course of the disease are directed toward control of fasciculations and muscle cramps. Mild muscle twitches are often treated with vitamin E or magnesium [25, 35]. However, as the cramps progress in intensity and duration, carbamazepine, quinine sulphate, or phenytoin may also be given [35]. With time and the development of spasticity, myorelaxants such as baclofen, a *γ*-amino-butyric acid (GABA) analog that facilitates spinal motor neuron inhibition, are employed [37, 38]. Oral baclofen is usually administered 2-3 times a day in a 10 mg dose, but can be titrated up to a 4 times per day—20 mg dose if necessary [36, 39]. Higher doses can produce problematic side effects such as sedation, weakness, and fatigue [36]. For these reasons, baclofen is often administered intrathecally to evade these adverse reactions. Other drugs used to treat spasticity in ALS include tizanidine, dantrolene sodium, diazepam, and memantine [25, 35, 39, 40]. Moreover, a combination of drugs may also be administered considering the unique mechanistic properties of these pain therapeutics. Although efficacious in offering some degree of symptomatic relief for pain, it is also necessary to mention that like baclofen, in excess, these myorelaxants can increase muscle weakness, further complicating the disease process in ALS. 

As the disease progresses and mobility decreases, pain becomes more common due to altered tone around joints, stiffness, and atrophy. To treat pain in advanced stages of ALS, nonsteroid anti-inflammatory drugs (NSAIDS) may also be given for moderate to severe pain. If necessary, narcotic analgesics are administered to achieve analgesia. In a hospice study where more than 80% of the patients received the therapy at least once a day, opioids effectively offered benefit to about 70% of the patients with advanced motor neuron disease [41, 42]. Despite these analgesic effects, opioids are associated with a number of side effects that can dramatically complicate ALS characteristic conditions. Narcotics can depress respiration, decrease airway protection, suppress cough, obstruct defecation, cause sedation, or result in physical dependence. Nevertheless, historically they have been the most efficacious agents used to offer meaningful relief in conditions of intractable pain. However, due to unwanted side effect attention has now been turned towards therapies that offer focal delivery of agents known to modulate the nociceptive cascade. In fact, intramuscular botulinum toxin (BoNT) injections have been used to reduce spasticity in ALS despite skepticism concerning muscle delivery in cases of continual muscle wasting [43, 44]. Yet, these new approaches to nociceptive control offer substantial promise for ALS pain. 

## 4. Gene Therapy as a Potential Therapeutic for ALS Pain

The use of intramuscular injections of BoNT for temporary pain relief in ALS has set the stage for the evaluation of *lasting* therapies to minimize ALS-associated pain conditions and revolutionized the treatment of focal spasticity. These studies demonstrated that focal injection of the Clostridial toxin into a pathologic microenvironment is still effective in combating aberrant nociceptive signaling despite persistent muscle wasting. Nevertheless, certain challenges still remain relevant to BoNT intramuscular administration in ALS patients. In particular, the transient nature of BoNT, lasting only a few months, creates a need for repeat application. Of greater concern is the observation of generalized weakness following BoNT administration in isolated cases [43–45]. Collectively, these studies suggest a need for more impressive means of modulating ALS pain transmission. Nevertheless, these studies present the argument for finding ways to stabilize BoNT expression to produce lasting results. 

Of the most exciting technologies that could be used to achieve lasting results is the employment of gene therapy to facilitate antinociceptive transgene expression. Gene therapy often involves the use of viral vectors to drive robust expression of a gene of interest. The gene is flanked by regulatory elements necessary for transcription and promoters that can be optimized to drive gene expression in restricted cell types or at selected time points. In the context of pain, a number of transgenes have been evaluated to regulate pain pathogenesis. Gene expression in these studies has involved the use of vectors derived from adenovirus, adenoassociated virus (AAV), lentivirus as well as herpes-simplex virus (HSV) [46, 47]. The unique tropism, cloning capacity and expression profiles of these vectors determine their ability to effectively modulate nociceptive signaling. Although a wide body of literature exists describing the use of viral vectors for conditions of chronic pain, little attention has been paid to how this is related in the context of ALS-pain [10, 11, 48–53]. Therefore, it is necessary to establish translational links between therapies shown to be effective in ALS pain and how targeted gene expression using agents known to mediate pain perception and transmission could offer substantial benefit in ALS-related nociception. 

### 4.1. Potential Transgenes

GABA is a major inhibitory neurotransmitter and the use of the GABA analog baclofen is the foremost therapy for ALS spasticity [35, 36, 39]. Although ALS-associated spasticity can be adequately controlled with baclofen, as stated earlier, disease progression requires increased dosage that can result in drug tolerance. Moreover, the use of implanted pumps for continual drug delivery carries the risk of infection, complication, or malfunction. Therefore, the use of viral vectors for one time administration of transgenes that result in GABA overproduction can have substantial advantages over currently used approaches.

One of the most widely studied genes for GABA overproduction is glutamic acid decarboxylase (GAD). The rate-limiting enzyme required for GABA production is GAD, which converts glutamate to GABA. Viral vector-driven GAD expression has been shown to have antinociceptive effects. Preclinical studies have demonstrated the benefits of gene delivery of GAD for the attenuation of pain in rodents using adenoviral, AAV, and HSV vectors [54–56]. This approach seems feasible for human application considering the clinical trials centered on the application of AAV-GAD for the treatment of overexcitation due to Parkinson disease [57–59]. Accordingly, clinical grade HSV vectors bearing GAD are currently being evaluated in rodent models of neuropathic pain [48, 53]. If successful, these studies could advance to clinical investigations into the safety and efficacy of HSV-GAD for attenuating pain in individuals with diabetic neuropathy [48]. Therefore, it is logical to assume that focal gene transfer of GAD could offer substantial benefit for spasticity in ALS.

The use of Clostridial neurotroxins has been shown to be beneficial for pain in studies involving focal delivery of BoNT [43–45]. However, this property could be greatly enhanced by coupling the control of ALS pain with viral vector technology. Gene delivery of BoNT could have lasting effects that evade the need for repeat administration. Alternatively, the use of bacterial toxins known to affect GABA transmission could also be just as beneficial. Specifically, our laboratory has demonstrated the use of the light chain (LC) fragment of the Clostridial tetanus neurotoxin in inhibiting synaptic function, thereby suppressing glutamatergic signaling [60]. Although these studies were not done in the context of pain, they demonstrated for the first time the benefits of viral vector driven LC expression to modulate synaptic activity in spinal motor neurons. Using adenoviral vectors to drive LC transgene expression, we were able to demonstrate these changes without neuronal cell death. We were also able to achieve substantial outcome measures indicating a profound influence on neurotransmitter release based on lumbar injections into the spinal cords of rats. We were also able to demonstrate that we could successfully affect the GABAergic system by adenoviral delivery of LC to the brain stem without altering surrounding CNS structures [60, 61]. Considering the brainstem derived pattern of activity that underlies painful spasticity in ALS patients, these studies suggest that an effective, neuronal specific approach such as this could be a viable approach for spasticity in ALS. 

Gene therapy also offers hope for musculoskeletal pain and pain associated with advanced ALS disease. Certain peripheral and spinal neurons are involved in mediating musculoskeletal pain. Muscle injury or joint trauma can increase nociceptive processing, and if inflammation ensues, peripheral nociceptors can become sensitized, resulting in increased neurotransmitter release in the dorsal horn of the spinal cord [62–65]. This sensitization often involves the activation of N-methyl-D-aspartate (NMDA) receptor signaling that leads to excitation of primary afferents at the site of injury, thereby potentiating the pain response [62, 63, 66]. These effects have been linked to conditions associated with the development and maintenance of arthritis [62, 63, 66–68]. Admittedly, arthritis is not a common condition found in the ALS population and cases where there is coexistence of the two disorders are probably due only to chance. However, an understanding of treatment approaches for chronic inflammatory pain conditions can provide valuable insight into how effective therapies can be applied to more acute pain conditions associated with impaired joint function in ALS. Interestingly, it has been shown that viral vector-mediated NMDA receptor elimination can decrease pain-like behaviors in mice [69]. Taken together, these studies suggest that ALS musculoskeletal pain can be attenuated by targeted inhibition of NMDA receptor signaling.

Opioids are the most commonly used treatment for pain in general. However, for ALS pain, opioids are not commonly prescribed until very late stages of the disease. Many opioid peptides exist. All of which result from one of three precursor peptides: proenkephalin-A, prodynorphin, or propiomelanocortin [70]. Proenkephalin-A, the only one found in the spinal cord, is responsible for producing the antinociceptive peptides met and leu-enkephalin. The anatomical distribution and receptor association properties of these peptides are responsible for the pain inhibitory properties of opiate drugs. 

Transgenic expression of opiate peptides has been shown to decrease pain behaviors in laboratory and clinical studies of chronic pain. Specifically, HSV-directed expression of proenkephalin has proven to be effective in attenuating both chronic and acute conditions using animal models of inflammatory, neuropathic, and bone cancer pain [53, 71–74]. Furthermore, a phase I study based on these preclinical findings is currently being conducted to evaluate the safety of a replication-defective HSV vector for effective delivery of preproenkephalin following intradermal vector delivery in patients with intractable cancer pain [48, 49]. If successful, these studies will allow for phase 2 trials aimed at determining the efficacy of HSV-mediated preproenkephalin in individuals with focal arthritic pain [48]. 

### 4.2. Therapeutic Application Considerations

Although gene therapy offers a practical approach to addressing pain in ALS, it is only a worthy pursuit if it has substantial advantages over commonly used pharmacologic strategies. Due to the lack of literature in the context of ALS describing investigations into pain incidence, effects on quality of life, origin and maintenance, or the tolerability and efficacy of drugs to circumvent pain syndromes in the ALS population, it is difficult to determine the specific problems associated with pain treatment in ALS. Nevertheless, an appreciation of the current issues associated with pain management in chronic disease offers substantial clues as to the criteria that a suitable alternative treatment approach must meet. 

A novel pain therapy for ALS would have to meet certain criteria. It would have to be safe and well tolerated with minimal off-target effects. It would be effective in modulating the nociceptive cascade to produce lasting effects, which will prevent the need for constant readministration of the therapy as is the case with pharmacologic agents. Gene therapy for pain in ALS should be adjustable and reversible. Several inducible systems have been developed to allow for regulated expression of transgenes [75–78]. To do so, the transgene of interest is expressed under the control of an inducible promoter that activates or represses transcription in the presence of biotic or abiotic factors. Such is the case with the tetracycline responsive promoter system where in the presence of doxycycline, promoter activity can be modulated to induce or hinder transgene expression due to its association with doxycycline and the tetracycline responsive transactivator protein complex. This system can allow for the regulated expression of antinociceptive transgenes as needed by the patient. Also, because doxycyline penetrates the blood-brain barrier and cerebral spinal fluid, it can be applied to control CNS gene expression as well [79, 80]. 

Careful consideration of delivery parameters is also important for determining if gene therapy is a suitable method for treating pain in ALS. Because recurrent pain usually suggests aberrant neural conduction properties in the spinal cord, treatment applications should involve a way to target spinal motor neurons. Direct spinal cord delivery of transgene, although risky, is a feasible treatment strategy. This approach carries with it additional challenges, however. There is the need for stable, long-term gene expression in that repeat administration would be impractical. There is also the concern that spinal cord injections could create further damage to an already toxic microenvironment due to surgery-associated spinal cord trauma. 

Remote delivery of therapeutic vectors may prove to have considerable advantages over direct spinal cord injection. Enthusiasm for muscle delivery of viral vectors for the retrograde delivery of therapeutic genes is centered on the fact that remote gene delivery of insulin-like growth factor 1 (Igf-1) has been shown to effectively achieve retrograde transport and increase survival in an animal model of ALS [81]. These results suggests that despite the die back of motor neurons, a pathological feature that has been associated with ALS, sufficient retrograde transport can still be achieved by the spared neural circuits that remain intact. 

Skeletal muscles are innervated by fibers from motor neurons. After peripheral inoculation, certain factors including Clostridial tetanus toxin are able to undergo retrograde transport to the CNS. It is important to note that the use of tetanus toxin for neuronal targeting presented here is not the same as that which has already been discussed in the context of its light chain fragment. Full-length tetanus toxin is composed of both a light and a heavy chain. Although the light chain is the means through which it exerts its protease activity, the heavy chain allows for cell binding and entry. Therefore, the coupling of the retrograde transport properties of the heavy chain with that of transgenes known to modulate nociception could prove to be an effective treatment approach for ALS pain. Thus, this could offer a means to target spinal cord neurons by muscle injection. 

Admittedly, considerable laboratory investigations into the generation and the use of these approaches have not been made. While the possibility of their application to the patient population affected by pain is a long way off, there are, however, alternative means for retrograde transport currently available. One of the unique features of HSV is that it has evolved a mechanism for retrograde transport. Moreover, these vectors can be produced to clinically relevant titers necessary for large-scale human therapy. Likewise, these vectors are currently being employed clinically in trials investigating potential ways to combat pain in advanced diseases [48, 49]. 

## 5. Conclusions

Pain in ALS is a commonly overlooked, understudied, underrated, and potentially undertreated aspect of the disease. This problem is not unique to ALS, however. This is due in large part to the traditional approaches that have been taken to evaluate pain in isolation of other pathologic conditions. This can have devastating effects on patients that greatly diminish quality of life, in that pain has been shown to be critical barrier to adequate care amongst the dying. In a study to investigate these concerns, family respondents of chronically ill individuals reported that at the time of death, patients experience moderate to severe pain [82]. Interestingly, this observation was highest amongst hospitalized individuals. Family respondents also identified that there was a need for more guidance and support to deal with the pain of the patient and nearly 30% of respondents believed that medical staff was reluctant to medicate [82]. These pain-related barriers to medical care echo earlier reports investigating pain among the elderly where nearly 50% of dying patients lack adequate pain treatment at the time of death [82, 83]. 

There are many reasons for the improper management of pain in ALS. It is the consequence of a number of factors that may include failure of the physician to recognize pain in the ALS patient. In order for the pain to be treated, it has to be reported. Since pain is not a primary consequence of the disease and not usually associated with ALS, routine pain assessments are seldom done. Another reason for inadequate pain management could be reluctance of the physician to administer pain medications. This could be out of fear of scrutiny from medical regulatory authorities as has been reported in studies investigating hospital staff response to increased reports of pain amongst the dying [84]. Moreover, patients lack the tendency to report pain in many cases. This could reflect a belief that pain is a normal aspect of the disease as has been the case with unreported pain amongst the elderly or individuals with cancer [85, 86]. Patient reluctance to report pain could also be derived out of fear that the implication of a pain treatment regimen might divert the physician's attention away from treatment of the primary consequences of the disease [86, 87]. This is devastating, considering pain is a highly treatable condition, and poor pain management only intensifies patient suffering and has drastic effects on the emotional and social well-being of ALS patients [7]. 

Proper pain management in ALS should involve a multidisciplinary approach just as is the case with other aspects of the disease. Considering half of ALS patients experience pain involving more than one type, no rigid treatment program that involves the sole use of a single agent should be employed to treat ALS-associated pain conditions [7]. Hence, a patient-specific approach should be taken to address pain in ALS palliative care. 

A number of therapies centered on modulation of the inhibitory GABAergic system have proven to be effective in treating ALS pain. Baclofen is widely used to treat spasticity, and its use is commonly implemented into the treatment plan during early stages of the disease. With disease progression, pain frequency and intensity can increase, creating the need for the use of narcotic agents. Opioids have proven to be effective in providing pain relief in advanced disease. Nevertheless, these therapies lack the ability to induce long-term lasting effects without constant administration. 

Gene therapy for pain associated with ALS could have substantial promise. Hallmark studies demonstrate the ability of viral vectors to attenuate pain by modulating inhibitory regulatory systems. Moreover, in advanced disease states, targeted gene delivery of opiate peptides can be used to modulate ALS-associated nociception. Therefore, the use of viral vectors could prove to be quite advantageous for treating pain, setting the stage for a new class of drugs effective at alleviating conditions observed in patients with ALS.
